# Melting of micro/nanoparticles considering anisotropy of surface energy

**DOI:** 10.1038/s41598-021-98704-3

**Published:** 2021-09-29

**Authors:** C. M. Yang, M. W. Chen, G. J. Zheng, Z. D. Wang

**Affiliations:** 1 School of Mathematics and Physics, University of Science and Technology, Beijing, Beijing, 100083 China; 2 School of Materials Science and Engineering, University of Science and Technology, Beijing, Beijing, 100083 China

**Keywords:** Materials science, Mathematics and computing, Nanoscience and technology, Physics

## Abstract

The effect of surface energy on the melting of micro/nanoparticles is studied using the asymptotic method. The asymptotic solution of the dynamic model for micro/nanoparticle melting reveals the dependence of the particle melting temperature on the particle size and the anisotropy of surface energy. Specifically, as the particle radius decreases, the isotropic surface energy reduces the melting temperature and accelerates the interface melting of the particle. Along certain crystal orientations, the anisotropy of surface energy enhances the melting temperature of the micro/nanoparticles, whereas depresses the melting temperature of the micro/nanoparticle along other crystal orientations. The anisotropy of surface energy enhances the melting speed of the micro/nanoparticles along certain crystal orientations, whereas reduces the melting speed of the micro/nanoparticles along other crystal orientations. The result of the asymptotic solution is in good agreement with the experimental data.

## Introduction

The melting of micro/nanoparticles initiates at the particle surface, and is greatly impacted by surface energy. The size dependence of melting temperature reduction has been verified experimentally for a number of different metals including In^1–7^, Pb^1^, Bi^1^, Al^8,9^, Sn^1,10^ (see Mei and Lu^11^ for a more complete summary of experimental results). Samsonov et al.^12–14^ found the size dependence of the melting temperature reduction for Al, Sn and Cu nanoparticles when the surface tension (surface energy) effect is considered in thermodynamic models and the melting temperature of nanoparticles is determined by thermodynamic factors. Dippel et al.^2^ and Lai et al.^8^ revealed that this melting behavior is originated from the positive solid–liquid surface energy from the thermodynamic way for the self-assembled Al nanoparticles. With a model without any free parameter, Jiang et al.^15–20^ found the size dependence of melting temperature reduction of Au, Sn, Al and Ag nanoparticles, and pointed out that the size dependence of melting temperature of the nanoparticles mainly depends on the ratio of the grain boundary energy to the surface energy. Ohashi et al.^3^ and Saka et al.^4^ investigated the melting of In nanoparticles embedded in Al and Fe matrices, and found that the melting temperature reduction strongly depends on the interface energy between the liquid In and the Fe matrix. Sasaki and Saka^5^ observed the melting process of In nanoparticles embedded in an Al matrix by using in-situ high-resolution transmission electron microscopy and revealed that the In nanoparticles have an orientation dependence with the Al matrix and the melting of different crystal surfaces occurs at different temperatures. Zhang and Cantor^6^ experimentally found that the melting of In nanoparticles in melt-spun hypomonotectic A1-7 wt% In exhibits an orientation relationship with the A1 matrix. In particular, the In nanoparticle melting temperature varied with the anisotropy of the Al–In surface energy, where the {100}_Al_ surface energy was on average 36% greater than the {111}_Al_‖{111}_ln_ surface energy and 25% greater than the {111}_Al_ surface energy. In addition to the experimental research, theoretical studies on the particle size dependence of the nanoparticles melting temperature have made significant progress. McCue et al.^21^ and Back et al.^22,23^ investigated the melting of a spherical particle by solving a two-phase Stefan problem with the Gibbs–Thomson condition. They found that surface energy reduces the melting temperature of solid nanoparticles with decreasing particle radius. In the present work, we study the melting of micro/nanoparticles while considering anisotropy of surface energy. We include the anisotropy of surface energy in the Gibbs–Thomson condition of the dynamic model for melting a micro/nanoparticle. By using the asymptotic method, we find the asymptotic solution of the temperature and interface speed of the micro/nanoparticles and reveal the dependence of the melting temperature on size, crystal orientation and interface speed of the micro/nanoparticles.

## Results

We consider a single nanoparticle of radius $R_{I}^{ * }$ and the temperature at infinity held at a constant $T_{\infty }^{ * }$ for all time. At the time $t^{ * } = 0$, the temperature on the liquid–solid interface $R^{ * } = R_{I}^{ * }$ is above the melting point, the particle will begin to melt. The interface function is represented by $R^{ * } = R^{ * } (\theta ,\varphi ,t^{ * } )$ and grows from $R^{ * } = R_{I}^{ * }$ towards the centre of the particle, where $\theta$, $\varphi$ are the azimuthal and polar angles in the spherical coordinate system whose center is the origin.

The problem is to solve for the temperature distributions $T_{L}^{ * }$ and $T_{S}^{ * }$ in the liquid and solid phases, respectively, as well as the interface function $R^{ * } = R^{ * } (\theta ,\varphi ,t^{ * } )$. As we are assuming that heat diffuses through both liquid and solid phases via conduction, the governing equations are that

1 $$\frac{{\partial T_{L}^{ * } }}{{\partial t^{ * } }} = \kappa_{L} \nabla^{2} T_{L}^{ * } ,\quad R^{ * } (\theta ,\varphi ,t^{ * } ) < r^{ * } < \infty ,$$

2 $$\frac{{\partial T_{S}^{ * } }}{{\partial t^{ * } }} = \kappa_{S} \nabla^{2} T_{S}^{ * } ,\quad 0 < r^{ * } < R^{ * } (\theta ,\varphi ,t^{ * } ),$$

where $\kappa_{L}$ and $\kappa_{S}$ are the thermal diffusivities in the liquid and solid phases, respectively. $\nabla^{2}$ is the Laplacian operator. At the interface $R^{ * } = R^{ * } (\theta ,\varphi ,t^{ * } )$, the temperature is continuous between the liquid and solid phases

3 $$T_{L}^{ * } = T_{S}^{ * } .$$

The Gibbs–Thomson condition holds that,

4 $$T_{I}^{ * } = T_{M}^{ * } \left( {1 + \frac{\gamma }{\Delta H}SK^{ * } } \right) - \frac{1}{\mu }U_{I}^{ * } ,$$

where $T_{I}^{ * }$ and $T_{M}^{ * }$ are the temperatures at the interface and the bulk melting point, respectively. $\gamma$ is the surface energy, $K^{ * }$ is the mean curvature at the interface, $S$ is the stiffness of the interface, $\Delta H$ is the latent heat per unit volume. $U_{I}^{ * }$ is the local interface speed. $\mu$ is the surface attachment kinetics coefficient.

At the interface we have the energy conservation condition

5 $$\Delta HU_{I}^{ * } = (k_{S} \nabla T_{S}^{ * } - k_{L} \nabla T_{L}^{ * } ) \cdot {\mathbf{n}},$$

where the thermodynamic constants $k_{L}$ and $k_{S}$ are respectively the heat conduction coefficients in the liquid and solid phases, $\nabla$ is the Hamiltonian operator, ${\mathbf{n}}$ is the unit vector normal to the interface.

The far-field temperature condition is that

6 $$\,T_{L}^{ * } \to T_{\infty }^{ * } > T_{M}^{ * } \quad {\text{as}}\,\,r^{ * } \to \infty .$$

The initial condition for the interface is written as

7 $$R^{ * } (\theta ,\varphi ,0) = R_{I}^{ * } \quad {\text{at}}\,\,t^{ * } = 0.$$

It proves particularly insightful to scale the problem using the following dimensionless quantity transformation

$$\begin{gathered} r = \frac{{r^{ * } }}{{R_{I}^{ * } }},\quad U_{I} { = }\frac{{U_{I}^{ * } }}{{V_{P} }},\quad t = \frac{{t^{ * } }}{{R_{I}^{ * } /V_{P} }}, \hfill \\ \hfill \\ \end{gathered}$$

8 $$T_{L} = \frac{{T_{L}^{ * } - T_{M}^{ * } }}{{\Delta H/(c_{pL} \rho_{L} )}},\quad T_{S} = \frac{{T_{s}^{ * } - T_{M}^{ * } }}{{\Delta H/(c_{pL} \rho_{L} )}},\quad R{ = }\frac{{R^{ * } }}{{R_{I}^{ * } }},$$

where $R_{I}^{ * }$, $V_{P}$, $R_{I}^{ * } /V_{P}$ and $\Delta H/(c_{pL} \rho_{L} )$ are length, speed, time and temperature scales, respectively. $V_{P} { = }k_{L} \Delta T/(R_{I}^{ * } \Delta H)$, $\Delta T = T_{\infty } - T_{M}^{ * }$. $\rho_{L}$ is the density in the liquid phase. $c_{\rho L}$ is the specific heat coefficient in the liquid phase. Equations (1)–(2) for the temperature fields in the liquid and solid phases are transferred into the following model.

The governing equations are that

9 $$\varepsilon \frac{{\partial T_{L} }}{\partial t} = \nabla^{2} T_{L} ,\quad R(\theta ,\varphi ,t) < r < \infty ,$$

10 $$\varepsilon \lambda_{T} \frac{{\partial T_{S} }}{\partial t} = \nabla^{2} T_{S} ,\quad 0 < r < R(\theta ,\varphi ,t),$$

which are subject to the following dimensionless boundary conditions.

At the interface $R = R(\theta ,\varphi ,t)$, the temperature is continuous between the liquid and solid phases

11 $$T_{L} = T_{S} ,$$

the Gibbs–Thomson condition holds that

12 $$T_{L} = \varepsilon \Gamma KS - \varepsilon ME^{ - 1} U_{I} ,$$

and the energy conservation condition holds that

13 $$\varepsilon U_{I} = (k_{T} \nabla T_{S} - \nabla T_{L} ) \cdot {\mathbf{n}}.$$

The far-field condition holds that

14 $$T_{L} \to \varepsilon \quad {\text{as}}\,\,r \to \infty .$$

The initial condition for the interface is that

15 $$R(\theta ,\varphi ,0) = 1\quad {\text{at}}\,\,t = 0.$$

Here, the dimensionless parameters are defined as the following notations

$$\varepsilon = \frac{\Delta T}{{\Delta H/(c_{\rho L} \rho_{L} )}},\quad \lambda_{T} = \frac{{\kappa_{L} }}{{\kappa_{S} }},\quad k_{T} = \frac{{k_{S} }}{{k_{L} }},\quad \kappa_{S} = \frac{{k_{S} }}{{c_{\rho S} \rho_{S} }},$$

16 $$\kappa_{L} = \frac{{k_{L} }}{{c_{\rho L} \rho_{L} }},\quad \Gamma = \frac{{\gamma T_{M}^{ * } }}{{R_{I}^{*} \Delta H\Delta T}},\quad M = \frac{{V_{P} }}{{\mu T_{M}^{ * } }},\quad E = \frac{\Delta T}{{T_{M}^{ * } }},$$

where $\varepsilon$ is a dimensionless relative superheating parameter. $\lambda_{T}$ is the ratio of the thermal diffusivities of the liquid and solid phases. $k_{T}$ is the ratio of the thermal conductivities of the solid and liquid phases. $c_{\rho S}$ is the specific heat parameter in the solid phase. $\rho_{S}$ is the density in the solid phase. $\Gamma$ is the surface energy parameter. $E$ is the relative superheating parameter, $M$ is the interfacial kinetics parameter. By nondimensionalizing our problem (1)–(7) according to the transformation (8), the resulting dimensionless parameters in (16) can be determined.

## Numerical Results

By using the asymptotic method, we solve the model in Eqs. (9)–(15) for the melting of a single micro/nanoparticle. The asymptotic solution of the temperature in the liquid and solid phases, the interface speed, and the interface function are expressed as

17 $$T_{L} = \varepsilon T_{L0} + \varepsilon^2 \left( {T_{L1^*} + T_{L1} } \right) + O\left( {\varepsilon^{3} } \right),\quad T_{S} = \varepsilon T_{S0} + \varepsilon^{2} \left( {T_{S1^*} + T_{S1} } \right) + O\left( {\varepsilon^{3} } \right),$$

18 $$\frac{\partial R}{{\partial t}} = \frac{{dR_{0} }}{d t} + \varepsilon \frac{{\partial R_{1} }}{\partial t} + O\left( {\varepsilon^{2} } \right),\quad R = R_{0} + \varepsilon R_{1} + O\left( {\varepsilon^{2} } \right).$$

A detailed representation of the asymptotic solution is shown in the “Methods” section.

To illustrate our theoretical results, we used the physical parameters of pure indium and aluminum nanoparticles in Table 1. The parameters of specific heat of solid and liquid phases of indium particles are from the open material property data (website is http://www.matweb.com/#opennewwindow).

**Table 1:** Approximate physical parameter values for In and Al.

	T_M_(K)	k_L_(Wm^-1^ K^-1^)	k_S_(Wm^-1^ K^-1^)	$\rho_{L}$ (kg m^-3^)	$\rho_{S}$ (kg m^-3^)	$c_{\rho L}$ (kg K^-1^)	$c_{\rho S}$ (kg K^-1^)	$\Delta H$ × 10^8^ (J m^-3^)	$\Delta T$ (K)
In	429^2^	236^24^	248^24^	7020^2^	7310^2^	843.15	506.15	2.833^2^	1.6
Al	933^22^	99^22^	236^22^	2385^22^	2700^22^	897^22^	1080^22^	10.67^22^	3.6

The solid–liquid phase specific heat parameters of indium particles are derived from open material property data.

**Figure 1:**
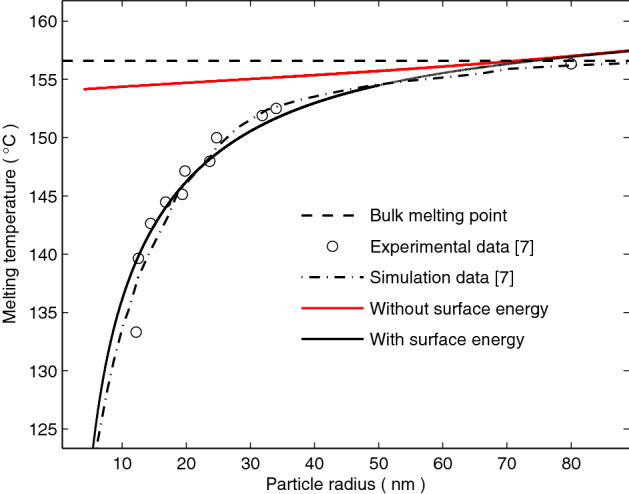
Size dependence of the melting temperature profile for indium nanoparticles. The black circles and black dotted line are the experimental data of a ball-milled sample and simulation data made by Lu and Jin^7^, respectively. The black solid line and red solid line denote respectively the melting temperatures with surface energy and without surface energy, plotted with the asymptotic solution (17). By contrast, the black dashed line denotes the bulk melting point.

Figure 1 shows the comparison of the size dependence of the melting temperature reduction of indium nanoparticles obtained from the experimental data and simulation data made by Lu and Jin^7^ with the asymptotic solution (17). In our asymptotic solution, when the surface energy is neglected (solid red line in Fig. 1, $\gamma_{0} = 0$), the size dependence of the melting temperature reduction of indium nanoparticles is lower than that of the bulk melting point (the black dashed line in Fig. 1). When surface energy is considered (solid black line in Fig. 1, $\gamma_{0} = 0.21$ J m^−2^) the predictions of the asymptotic solution agree very well with the experimental data^7^, only exhibiting a slight deviation for $20 < R < 40$ nm. This suggests that the deviation of the size dependence of the melting temperature reduction is caused by the anisotropic effect of surface energy.

**Figure 2:**
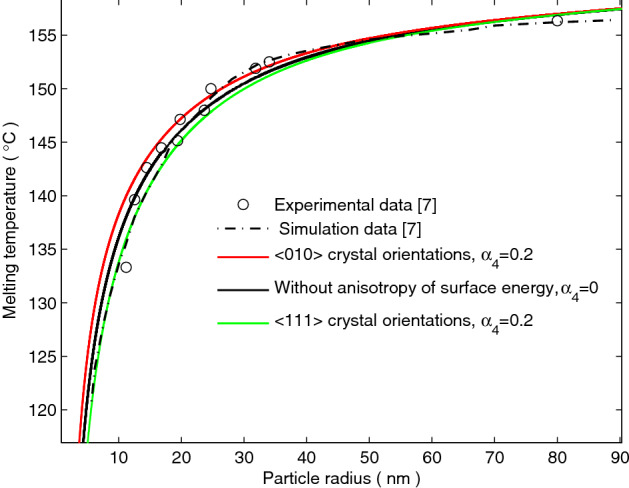
Size dependence of the melting temperature profile for indium nanoparticles. The black circles and black dotted line are the experimental data of a ball-milled sample and simulation data made by Lu and Jin^7^, respectively. The green and red lines denote the melting temperatures, plotted with the asymptotic solution (17) when the anisotropy of surface energy is considered. By contrast, the black solid line denotes the size dependent melting temperature reduction when disregarding the anisotropy of surface energy.

Figure 2 shows the size dependence of melting temperature reduction of indium nanoparticle which corresponds to the experimental data made by Lu and Jin^7^. When the anisotropy of surface energy is disregarded, the curve of the asymptotic solution (17) has a significant deviation to the experimental data in the range of more than 20 nm and less than 40 nm for indium nanoparticle. When the anisotropy of surface energy is considered, the curve of the asymptotic solution corresponds to more experimental data for indium nanoparticle along the <010> crystal orientations (red solid line in Fig. 2, $\alpha_{4} = 0.2$).

**Figure 3:**
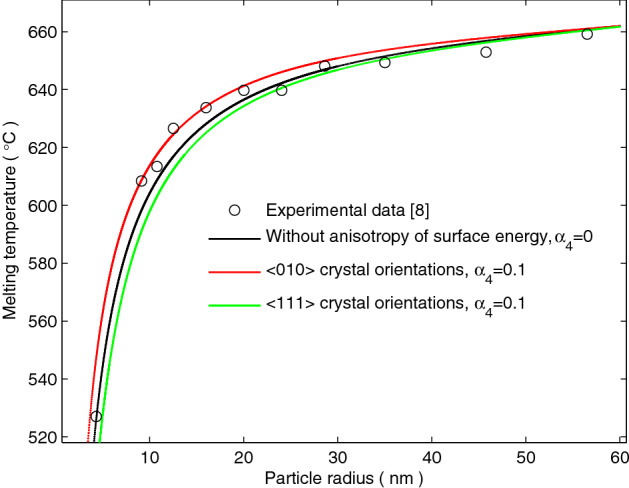
Size dependence of the melting temperature profile for Al nanoparticles. The black circles are the experimental data of a self-assembled Al nanoparticles made by Lai et al.^8^. The red and green lines represent the melting temperatures along the <010> and <111> crystal orientations, plotted with the asymptotic solution (17) considering the anisotropy of surface energy.

Figure 3 plots the size dependence of the melting temperature reduction of Al nanoparticle which corresponds to the experimental data made by Lai et al.^8^. When the anisotropy of surface energy is disregarded, the curve of the asymptotic solution has a significant deviation to the experimental data in the range of more than 15 nm and less than 25 nm for Al nanoparticle. When the anisotropy of surface energy is considered in the asymptotic solution (17), the curve of the asymptotic solution corresponds to more experimental data for Al nanoparticle along the <010> crystal orientations.

From Figs. 2 and 3, it can be seen that the size dependence of the melting temperature reduction of the micro/nanoparticles depends not only on the isotropic surface energy, but also on the anisotropy of surface energy.

When the anisotropy of surface energy is considered, it is seen in Figs. 2 and 3 that the anisotropy of surface energy enhances the melting temperature of the micro/nanoparticles along the <010> crystal orientations, whereas depresses the melting temperature of the micro/nanoparticles along the <111> crystal orientations. The experimental results of Zhang and Cantor^6^ confirmed that the melting temperature varies with the anisotropy of surface energy and exhibits a dependence relationship with the crystal orientations.

**Figure 4:**
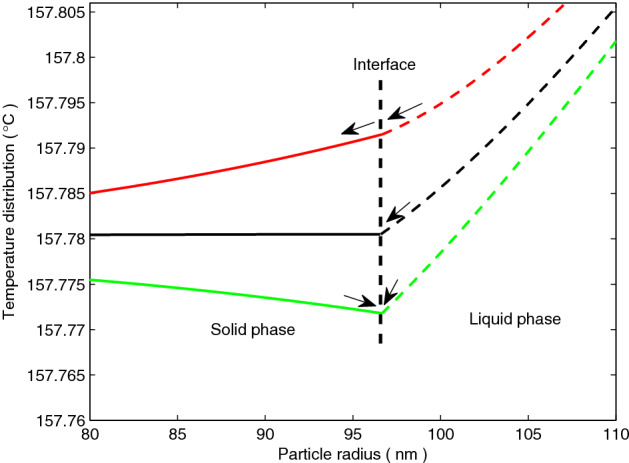
Temperature distributions of indium nanoparticle in the liquid phase (dashed lines) and the solid phase (solid lines) near the interface (vertical dashed line) along the < 010> (red lines) and <111> (green lines) crystal orientations. By contrast, the black solid and dashed lines denote the temperature distribution without the anisotropy of surface energy in the asymptotic solution (17). The arrows represent the heat flux directions.

Figure 4 shows the temperature distributions in the solid and liquid phases near the interface for indium nanoparticles, which are affected by the anisotropy of surface energy corresponding to Fig. 2. It is seen that the temperature gradients for the liquid and solid phases near the interface (vertical dashed line in Fig. 4) are different along the <111> (green lines in Fig. 4) and <010> (red lines in Fig. 4) crystal orientations. Along the <111> crystal orientations, there feature the temperature gradients ${{\partial T_{L} } \mathord{\left/ {\vphantom {{\partial T_{L} } {\partial r(R,t)}}} \right. \kern-\nulldelimiterspace} {\partial r(R,t)}} > 0$ and ${{\partial T_{S} } \mathord{\left/ {\vphantom {{\partial T_{S} } {\partial r(R,t)}}} \right. \kern-\nulldelimiterspace} {\partial r(R,t)}} < 0$ near the interface, signifying that the heat flux flows from both the solid and liquid phases into the interface (arrows on green lines in Fig. 4). The temperature in the solid phase is always higher than at the interface, and the heat flux flows out from the solid phase into the interface. This behavior reduces the equilibrium temperature at the interface and then reduces the melting temperature. Along the <010> crystal orientations, there exist the temperature gradients ${{\partial T_{L} } \mathord{\left/ {\vphantom {{\partial T_{L} } {\partial r(R,t)}}} \right. \kern-\nulldelimiterspace} {\partial r(R,t)}} > 0$ and ${{\partial T_{S} } \mathord{\left/ {\vphantom {{\partial T_{S} } {\partial r(R,t)}}} \right. \kern-\nulldelimiterspace} {\partial r(R,t)}} > 0$ near the interface, signifying that the heat flux flows into the interface from the liquid phase and then flows into the solid phase from the interface (arrows on red lines in Fig. 4). Along the <010> crystal orientations, in addition to the heat flux required for melting, the surplus of heat flux then flows from the interface into the solid phase. While heat flux is flowing into the solid phase from the interface, the melting temperature increases with the increasing equilibrium temperature at the interface. When the anisotropy of surface energy is neglected (black line in Fig. 4), there feature the temperature gradients ${{\partial T_{L} } \mathord{\left/ {\vphantom {{\partial T_{L} } {\partial r(R,t)}}} \right. \kern-\nulldelimiterspace} {\partial r(R,t)}} > 0$ and ${{\partial T_{S} } \mathord{\left/ {\vphantom {{\partial T_{S} } {\partial r(R,t)}}} \right. \kern-\nulldelimiterspace} {\partial r(R,t)}} = 0$ near the interface, signifying that there is no heat flux from the interface into the solid phase. This explains that compared with the isotropic surface energy, the anisotropy of surface energy enhances the melting temperature of micro/nanoparticles along the <010> crystal orientations, whereas depresses the melting temperature of micro/nanoparticles along the <111> crystal orientations. The differences in the temperature gradients of two phases near the interface affected by the anisotropy of surface energy results in the differences in the heat flux direction during the melting process, which explains the dependence of melting temperature on crystal orientations observed in the experiment of Zhang and Cantor^6^.

**Figure 5:**
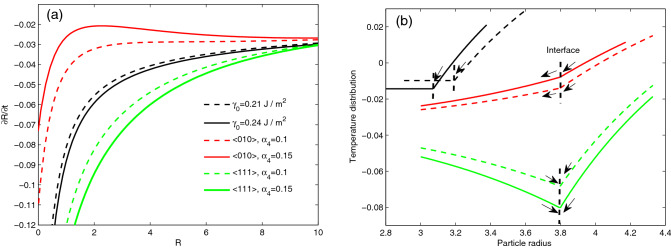
(**a**) Dimensionless dependence of the interface speed $\partial R/\partial t$ on the particle radius *R* under various surface energy values, plotted by the asymptotic solution (18). (**b**) Dimensionless temperature distributions in the solid and liquid phases near the interface (black dotted lines) corresponding to the value of surface energy in (**a**), plotted by the asymptotic solution (17). The temperatures of the solid and liquid phases are located on the left and right sides of the interface (vertical dashed lines), respectively. The arrows represent the heat flux directions.

Figure 5a illustrates the dimensionless dependence of the interface speed ${{\partial R} \mathord{\left/ {\vphantom {{\partial R} {dt}}} \right. \kern-\nulldelimiterspace} {\partial t}}$ on the particle radius $R$ under various surface energy values using the asymptotic solution (18). The negative interface speed indicates the direction of the particle radius moving towards the particle center. When the isotropic surface energy is considered, the interface speed monotonically increases with decreasing particle radius. When the isotropic surface energy increases from 0.21 to 0.24 J m^−2^, the interface speed increases further, with a more notable increase for smaller values of $R$. When the anisotropy of surface energy is considered, compared with considering the isotropic surface energy (black line in Fig. 5a), along the <111> (green lines in Fig. 5a) crystal orientations the interface speed is greater, while the interface speed along the <010> (red lines in Fig. 5a) crystal orientations is lower. The interface speed along the <111> crystal orientations is faster than that along the <010> crystal orientations. According to the heat flux directions along the <111> and <010> crystal orientations plotted in Fig. 4, it is the difference of the heat flux direction that induces the different trend of the interface speed along two crystal orientations. An increase of the anisotropy of surface energy (from $\alpha_{4} = 0.1$ to $\alpha_{4} = 0.15$) increases the interface speed along the <111> crystal orientations, while decreases the interface speed along the <010> crystal orientations.

In Fig. 5b, we show the dimensionless temperature distributions of the two phases corresponding to the surface energy in Fig. 5a. The negative dimensionless temperature indicates that the temperature affected by the surface energy shown in Fig. 5a is lower than the bulk melting point. Figure 5b shows that an increase of the surface energy results in a large change in the temperature gradients of the two phases near the interface. The surface energy increases from 0.21 J m^−2^ (black dashed line in Fig. 5b) to 0.24 J m^−2^ (black solid line in Fig. 5b) in the asymptotic solution (17), which enhances the temperature gradient of the liquid phase near the interface, causing more heat flux from the liquid phase into the interface. Along the <010> crystal orientations, the increased anisotropy of surface energy reduces the temperature gradient of the liquid phase near the interface, whereas the temperature gradient of the solid phase near the interface is enhanced. Therefore, the increased anisotropy of surface energy causes more surplus heat flux from the interface into the solid phase along those crystal orientations. Along the <111> crystal orientations, the increased anisotropy of surface energy enhances the temperature gradient of the liquid phase near the interface, whereas the temperature gradient of the solid phase near the interface is reduced. This means that the increased anisotropy of surface energy causes more heat flux into the interface from the liquid and solid phases along those orientations. Consequently, along certain crystal orientations, the increased anisotropy of surface energy enhances the interface speed and thus speeds up the melting process, whereas reduces the interface speed and thus slows down the melting process along other crystal orientations.

It is noteworthy that, along the <111> crystal orientations, the temperature in the solid phase is always higher than the melting temperature near the interface. The solid phase undergoes superheating as local phenomenon, which could be referred to as local superheating. Font and Myers^25^ pointed out that the superheating is owing to the melting temperature at the interface decreasing faster than the heat diffusion in the solid phase. McCue et al.^21^ suggested that the solid is locally superheated after a point in time as the melting temperature decreases. We suggest that the micro/nanoparticles along the <111> crystal orientations undergo some form of local superheating during the melting process, which is revealed when the anisotropy of surface energy is taken into consideration. With the increase of anisotropy of surface energy, more heat flux flows into the interface from the solid phase, thus reducing the superheating temperature. In addition, the interface speed along those crystal orientations is higher than other crystal orientations.

## Discussion

In this paper, we study the melting of micro/nanoparticles possessing the anisotropy of surface energy. The anisotropy of surface energy is included in the Gibbs–Thomson condition of the dynamic model for micro/nanoparticle melting. Using the asymptotic method, we find the asymptotic solution of the temperature and interface speed of micro/nanoparticles of varying size, and reveal the dependence of the melting temperature on size and interface speed of the micro/nanoparticles.

When the surface energy is considered, the curve of the asymptotic solution has a significant deviation to the experimental data for In and Al nanoparticles. When the anisotropy of surface energy is considered, the curve of the asymptotic solution corresponds to more experimental data for In and Al nanoparticle along the <010> crystal orientations. The size dependence of the melting temperature reduction of nanoparticles depends not only on the isotropic surface energy, but also on the anisotropy of surface energy. Also, our asymptotic solution shows the size dependence of the melting temperature when the surface energy is not considered.

When the anisotropy of surface energy is taken into consideration, the asymptotic solution reveals the fundamental cause underlying Zhang and Cantor's experimental observations that the melting temperature varies with the anisotropy of surface energy^6^. The differences in the temperature gradients of the liquid and solid phases near the interface affected by the anisotropy of surface energy result in the differences in the heat flux direction during the melting process, which is the mechanism behind the variations of melting temperature with different crystal orientations.

As the heat flux is flowing into the interface from the liquid phase, the melting process accelerates rapidly, leading to a dramatic increase in interface speed. The increase of the surface energy increases the interface speed, where the increase is most notable for smaller particles. When the anisotropy of surface energy is considered, the crystal orientations wherein the heat flux flows out from the solid phase exhibit a greater interface speed than the crystal orientations wherein the heat flux surplus flows into the solid phase. Along certain crystal orientations, the increased anisotropy of surface energy promotes the heat flux flows into interface from the solid phase, thus speeds up the melting process, whereas along other crystal orientations, the increased anisotropy of surface energy depresses the heat flux surplus flows into the solid phase from interface, thus slows down the melting process.

When the anisotropy of surface energy is considered, the temperature in the solid phase is higher than the melting temperature near the interface along some crystal orientations. This is referred to as local superheating of the solid phase. Previous reports^21,25^ explained this phenomenon based on various considerations. Excepting the melting process and the melting temperature, little is known about the properties of superheated particles. The results presented in this paper suggest that the increased anisotropy of surface energy reduces the superheating temperature and increases the melting speed.

## Methods

The dimensionless problem in Eqs. (9)–(15) are solved by using the asymptotic method. According to the theory of solidification, energy transfer occurs mainly near the interface, but far from the interface relatively smooth. Mathematically, there are different scales near the interface and far from the interface for the whole melt region. With the slow variable $\overline{r} = \varepsilon r$ introduced,

$$\frac{\partial }{\partial r} \to \frac{\partial }{\partial r} + \varepsilon \frac{\partial }{{\partial \overline{r}}},$$

the multiple variables $r$,$\overline{r}$,$\theta$,$\varphi$ are viewed as independent variables. The interface function is expressed as

19 $$\overline{R} = \overline{R}_{0} + \varepsilon \overline{R}_{1} + \cdot \cdot \cdot ,$$

where $$\overline{R} = \varepsilon R$$, $$\overline{R}_{0} = \varepsilon R_{0}$$, $$\overline{R}_{1} = \varepsilon R_{1}$$.

The temperature fields in the liquid and solid phases (9)–(10) are transferred into the governing equations

20 $$\varepsilon \frac{{\partial T_{L} }}{\partial t} = \nabla^{2} T_{L} + 2\varepsilon \frac{{\partial^{2} T_{L} }}{{\partial r\partial \overline{r}}} + \varepsilon^{{2}} \frac{{\partial^{2} T_{L} }}{{\partial \overline{r}^{2} }} + \varepsilon \frac{2}{r}\frac{{\partial T_{L} }}{{\partial \overline{r}}},\quad R(\theta ,\varphi ,t) < r < \infty ,$$

21 $$\varepsilon \lambda_{T} \frac{{\partial T_{S} }}{\partial t} = \nabla^{2} T_{S} + 2\varepsilon \frac{{\partial^{2} T_{S} }}{{\partial r\partial \overline{r}}} + \varepsilon^{{2}} \frac{{\partial^{2} T_{S} }}{{\partial \overline{r}^{2} }} + \varepsilon \frac{2}{r}\frac{{\partial T_{S} }}{{\partial \overline{r}}},\quad 0 < r < R(\theta ,\varphi ,t),$$

which are subject to the following boundary conditions. At the interface,

22 $$T_{L} = T_{S} ,$$

23 $$T_{L} = \varepsilon \Gamma KS - \varepsilon E^{ - 1} MU_{I} ,$$

24 $$\varepsilon U_{I} = (k_{T} \nabla T_{S} - \nabla T_{L} ) \cdot {\mathbf{n}} + \varepsilon \frac{\partial }{{\partial \overline{r}}}(k_{T} T_{S} - T_{L} ).$$

The mean curvature at the interface $K$, the stiffness of the interface $S$ and the surface energy $\gamma$ in surface energy parameter $\Gamma$ are expanded as follows,

$$K = - \frac{{1}}{{R_{0} }} + \frac{\varepsilon }{{2R_{0}^{2} }}(\Lambda + 2)R_{1} + \cdots \quad \Lambda = \frac{{\partial^{2} }}{{\partial \theta^{2} }} + \cot \theta \frac{\partial }{\partial \theta } + \frac{1}{{\sin^{2} \theta }}\frac{{\partial^{2} }}{{\partial \varphi^{2} }},$$

$$S = 2 + \alpha_{4} \left( {\frac{6}{5} - \frac{36}{5}P_{4}^{0} (\cos \theta ) - \frac{3}{70}P_{4}^{4} (\cos \theta )\cos 4\varphi } \right),\quad \gamma = \gamma_{0} [1 + \alpha_{4} (\sin^{4} \theta (\sin^{4} \varphi + \cos^{4} \varphi ) + \cos^{4} \theta )],$$

where $P_{n}^{m} (\cos \theta )\cos m\varphi$ is the spherical harmonic of degree $n$ and order $m$. $\gamma_{0}$ is the surface energy. $\alpha_{4}$ is the anisotropic parameter of surface energy.

The far-field temperature condition and the initial condition for the interface remain the same as (14) and (15).

Substituting Eqs. (17)–(18) into the above dimensionless problem (20)–(24), we have the equations for the each order term. The leading order terms $T_{L0}$, $T_{S0}$ and $R_{0}$ in Eqs. (20)–(21) satisfy the equations

25 $$\nabla^{2} T_{L0} = 0,\quad \nabla^{2} T_{S0} = 0,$$

which are subject to the boundary conditions: at the interface,

26 $$T_{L0} = T_{S0} ,$$

27 $$T_{L0} = - \frac{2\Gamma }{{R_{0} }} - E^{ - 1} M\frac{{dR_{0} }}{dt},$$

28 $$\frac{{dR_{0} }}{dt} = k_{T} \frac{{\partial T_{S0} }}{\partial r} - \frac{{\partial T_{L0} }}{\partial r}.$$

The far-field temperature condition is that

29 $$T_{L0} \to 1\,\,{\text{as}}\,\,r \to \infty ,\quad \overline{r} \to \infty ,$$

The initial condition for the leading order interface is that

30 $$R_{0} (0) = 1\,\,{\text{at}}\,\,t = 0,$$

The solution of Eq. (25), which obey the conditions (26)–(30), are expressed as

31 $$T_{L0} = 1 + \frac{{R_{0}^{2} }}{r}\frac{{dR_{0} }}{dt}e^{{\overline{R}_{0} - \overline{r}}} ,\quad T_{S0} = 1 + R_{0} \frac{{dR_{0} }}{dt},$$

where $R_{0}$ is the solution of the ordinary differential equation

$$\frac{{dR_{0} }}{dt} = - \frac{{R_{0} + 2\Gamma }}{{R_{0} (R_{0} + E^{ - 1} M)}},$$

which obeys the initial condition (30),

32 $$t = \frac{{1 - R_{0}^{2} }}{2} + (E^{ - 1} M - 2\Gamma )(1 - R_{0} ) + 2\Gamma (2\Gamma - E^{ - 1} M)\ln \frac{1 + 2\Gamma }{{R_{0} + 2\Gamma }}.$$

The first order terms $T_{L1}$, $T_{S1}$ and $R_{1}$ in Eqs. (20)–(21) satisfy the equations

33 $$\frac{{\partial T_{L0} }}{\partial t} = \nabla^{2} T_{L1} + 2\frac{{\partial^{2} T_{L0} }}{{\partial r\partial \overline{r}}} + \frac{2}{r}\frac{{\partial T_{L0} }}{{\partial \overline{r}}},$$

34 $$\lambda_{T} \frac{{\partial T_{S0} }}{\partial t} = \nabla^{2} T_{S1} + 2\frac{{\partial^{2} T_{S0} }}{{\partial r\partial \overline{r}}} + \frac{2}{r}\frac{{\partial T_{S0} }}{{\partial \overline{r}}},$$

which are subject to the boundary conditions:

35 $$T_{L1} = T_{S1} + \frac{{dR_{0} }}{dt}R_{1} + R_{0} \frac{{dR_{0} }}{dt}\overline{R}_{1} ,$$

36 $$T_{S1} = \frac{{(\Lambda + 2)\Gamma R_{1} }}{{R_{0}^{2} }} - E^{ - 1} M\frac{{\partial R_{1} }}{\partial t} - \frac{\Theta \Gamma }{{R_{0} }}\left( {\frac{6}{5} - \frac{36}{5}P_{4}^{0} (\cos \theta ) - \frac{3}{70}P_{4}^{4} (\cos \theta )\cos 4\varphi } \right),$$

37 $$\frac{{\partial R_{1} }}{\partial t} = k_{T} \frac{{\partial T_{S1} }}{\partial r} - \frac{{\partial T_{L1} }}{\partial r} - \frac{{2R_{1} }}{{R_{0} }}\frac{{dR_{0} }}{dt} - \frac{{dR_{0} }}{dt}\overline{R}_{1} + \frac{{dR_{0} }}{dt}R_{0} ,$$

where $\Theta$ is defined in $\alpha_{4} = \Theta \varepsilon$. The anisotropic parameter of surface energy $\alpha_{4}$ is assumed to be of the same order of magnitude as $\varepsilon$, $\Theta = O(1)$.

The far-field temperature condition holds that

38 $$T_{L1} \to 0\,\,{\text{as}}\,\,r \to \infty ,\quad \overline{r} \to \infty .$$

The initial condition for the first order interface is that

39 $$R_{1} (\theta ,\varphi ,0) = 0\,\,{\text{at}}\,\,t = 0$$

The particular solution of the first order terms in Eqs. (33)–(34) are expressed as

40 $$T_{{L1^{ * } }} = \frac{r}{2}\frac{d}{dt}\left( {R_{0}^{2} \frac{{dR_{0} }}{dt}} \right)e^{{\overline{R}_{0} - \overline{r}}} + \frac{{rR_{0}^{2} }}{2}\frac{{dR_{0} }}{dt}\frac{{d\overline{R}_{0} }}{dt}e^{{\overline{R}_{0} - \overline{r}}} ,\quad T_{{S1^{ * } }} = \frac{{\lambda_{T} r^{2} }}{6}\frac{d}{dt}\left( {R_{0} \frac{{dR_{0} }}{dt}} \right).$$

It is seen that the introduction of multiple scales guarantees that the solutions for the temperature field in (40) satisfy the far-field temperature condition (38). The solution of Eqs. (33)–(34), which obey the conditions (35)–(39), is expressed as

$$\begin{aligned} & T_{L1} = \frac{{A_{0,0} }}{r} + \frac{{A_{4,0} }}{{r^{5} }}P_{4} (\cos \theta ) + \frac{{A_{4,4} }}{{r^{5} }}P_{4}^{4} (\cos \theta )\cos 4\varphi , \\ & T_{S1} = B_{0,0} + B_{4,0} r^{4} P_{4} (\cos \theta ) + B_{4,4} r^{4} P_{4}^{4} (\cos \theta )\cos 4\varphi , \\ \end{aligned}$$

and the first order term of the interface *R*_1_ are expressed as

41 $$R_{1} = g_{0,0} + g_{4,0} P_{4} (\cos \theta ) + g_{4,4} P_{4}^{4} (\cos \theta )\cos 4\varphi ,$$

where $A_{m,n}$, $B_{m,n}$, $g_{0,0}$, $g_{4,0}$ and $g_{4,4}$ are determined by the interface conditions (35)–(37). For the mode $n = 0$, $m = 0$,

$$\begin{aligned} A_{0,0} & = R_{0} B_{0,0} + R_{0} \frac{{dR_{0} }}{dt}g_{0,0} + R_{0}^{2} \frac{{dR_{0} }}{dt}\overline{g}_{0,0} - \frac{1}{2}R_{0}^{2} \frac{d}{dt}\left( {R_{0}^{2} \frac{{dR_{0} }}{dt}} \right) \\ & \quad + \frac{{\lambda_{T} R_{0}^{3} }}{6}\frac{d}{dt}\left( {R_{0} \frac{{dR_{0} }}{dt}} \right) - \frac{{R_{0}^{4} }}{2}\frac{{dR_{0} }}{dt}\frac{{d\overline{R}_{0} }}{dt}, \\ B_{0,0} & = \frac{{2\Gamma g_{0,0} }}{{R_{0}^{2} }} - E^{ - 1} M\frac{{dg_{0,0} }}{dt} - \frac{{\lambda_{T} R_{0}^{2} }}{6}\frac{d}{dt}\left( {R_{0} \frac{{dR_{0} }}{dt}} \right) - \frac{6}{5}\frac{\Theta \Gamma }{{R_{0} }}; \\ \end{aligned}$$

for the mode $n = 4$, $m = 0$,

$$\begin{aligned} & A_{4,0} = R_{0}^{9} B_{4,0} + R_{0}^{5} \frac{{dR_{0} }}{dt}g_{4,0} + R_{0}^{6} \frac{{dR_{0} }}{dt}\overline{g}_{4,0} , \\ & B_{4,0} = - \frac{18\Gamma }{{R_{0}^{6} }}g_{4,0} - \frac{{E^{ - 1} M}}{{R_{0}^{4} }}\frac{{dg_{4,0} }}{dt} + \frac{36\Theta \Gamma }{{5R_{0}^{5} }}; \\ \end{aligned}$$

for the mode $n = 4$, $m = 4$,

$$\begin{aligned} & A_{4,4} = R_{0}^{9} B_{4,4} + R_{0}^{5} \frac{{dR_{0} }}{dt}g_{4,4} + R_{0}^{6} \frac{{dR_{0} }}{dt}\overline{g}_{4,4} , \\ & B_{4,4} = - \frac{18\Gamma }{{R_{0}^{6} }}g_{4,4} - \frac{{E^{ - 1} M}}{{R_{0}^{4} }}\frac{{dg_{4,4} }}{dt} + \frac{3\Theta \Gamma }{{70R_{0}^{5} }}, \\ \end{aligned}$$

where $g_{0,0}$, $g_{4,0}$ and $g_{4,4}$ satisfy the following ordinary differential equations respectively. $\overline{g}_{0,0} = \varepsilon g_{0,0}$, $\overline{g}_{4,0} = \varepsilon g_{4,0}$ and $\overline{g}_{4,4} = \varepsilon g_{4,4}$. For the mode $n = 0$, $m = 0$,

42 $$\begin{aligned} \frac{{dg_{0,0} }}{dt} & = \frac{1}{{R_{0}^{2} }}\Re (R_{0} ,0)g_{0,0} + \frac{1}{{D\left( {R_{0} ,0} \right)}}\left( {\frac{{\lambda_{T} k_{T} }}{3}R_{0}^{2} \frac{d}{dt}\left( {R_{0} \frac{{dR_{0} }}{dt}} \right)} \right.\left. { - R_{0}^{3} \frac{{dR_{0} }}{dt}\frac{{d\overline{R}_{0} }}{dt}} \right) \\ & \quad + \frac{1}{{D\left( {R_{0} ,0} \right)}}\left(  R_{0}^{2} \frac{{dR_{0} }}{dt}{ - R_{0} \frac{d}{dt}\left( {R_{0}^{2} \frac{{dR_{0} }}{dt}} \right)} \right.\left. { - \frac{6\Theta \Gamma }{{5R_{0} }}} \right); \\ \end{aligned}$$

for the mode $n = 4$, $m = 0$,

43 $$\frac{{dg_{4,0} }}{dt} = -\frac{3}{{R_{0}^{2} }}\Re (R_{0} ,4)g_{4,0} + \frac{36}{5}\frac{{\Gamma \Theta (4k_{T} + 5)}}{{R_{0} D(R_{0} ,4)}} + \frac{{4R_{0} }}{{D(R_{0} ,4)}}\frac{{dR_{0} }}{dt}\overline{g}_{4,0} ;$$

for the mode $n = 4$, $m = 4$,

44 $$\frac{{dg_{4,4} }}{dt} =-\frac{{3\Re (R_{0} ,4)}}{{R_{0}^{2} }}g_{4,4} + \frac{{3\Gamma \Theta (4k_{T} + 5)}}{{70R_{0} D(R_{0} ,4)}} + \frac{{4R_{0} }}{{D(R_{0} ,4)}}\frac{{dR_{0} }}{dt}\overline{g}_{4,4} ,$$

where $\Re (R_{0} ,n)$ and $D(R_{0} ,n)$ are two abbreviations,

$$\Re (R_{0} ,n) = \frac{1}{{D(R_{0} ,n)}}\left( {(n + 2)(nk_{T} + n + 1)\Gamma - R_{0}^{2} \frac{{dR_{0} }}{dt}} \right),\quad D(R_{0} ,n) = R_{0} + (nk_{T} + n + 1)E^{ - 1} M.$$

From the initial condition (39), it follows that $g_{0,0} (0) = 0$, $g_{4,0} (0) = 0$ and $g_{4,4} (0) = 0$. Then the solutions of Eqs. (42)–(44) are solved respectively. For the mode $n = 0$, $m = 0$,

45 $$\begin{aligned}&g_{0,0} = \frac{{\lambda_{T} k_{T} }}{{3H(R_{0} ,0)}}\int_{{R_{0} }}^{1} {\frac{{\tau^{2} H(\tau ,0)}}{D(\tau ,0)}\frac{d}{d\tau }\left( {\frac{2\Gamma + \tau }{{\tau + E^{ - 1} M}}} \right)} d\tau - \frac{1}{{H(R_{0} ,0)}}\int_{{R_{0} }}^{1} {\frac{{\tau^{2} H(\tau ,0)}}{D(\tau ,0)}\frac{{d\overline{R}_{0} \left( \tau \right)}}{d\tau }} d\tau \\ \quad + &\frac{6\Theta \Gamma }{{5H(R_{0} ,0)}}\int_{{R_{0} }}^{1} {\frac{H(\tau ,0)}{{D(\tau ,0)}}\frac{{\tau + E^{ - 1} M}}{2\Gamma + \tau }} d\tau +\frac{1}{{H(R_{0} ,0)}}\int_{{R_{0} }}^{1} {\frac{{\tau^{2} H(\tau ,0)}}{D(\tau ,0)}} d\tau \\\quad + &\frac{1}{{H(R_{0} ,0)}}\int_{{R_{0} }}^{1} {\frac{\tau H(\tau ,0)}{{D(\tau ,0)}}\frac{d}{d\tau }\left( {\frac{\tau (2\Gamma + \tau )}{{\tau + E^{ - 1} M}}} \right)} d\tau ; \\ \end{aligned}$$

for the mode $n = 4$, $m = 0$,

46 $$g_{4,0} = - \frac{{36\Gamma \Theta \left( {4k_{T} + 5} \right)}}{{5H\left( {R_{0} ,4} \right)}}\int_{{R_{0} }}^{1} {\frac{{H\left( {\tau ,4} \right)\left( {\tau + E^{ - 1} M} \right)}}{{D\left( {\tau ,4} \right)\left( {2\Gamma + \tau } \right)}}} d\tau + \frac{4}{{H\left( {R_{0} ,4} \right)}}\int_{{R_{0} }}^{1} {\frac{\tau H(\tau ,4)}{{D(\tau ,4)}}} \overline{g}_{4,0} d\tau ;$$

for the mode $n = 4$, $m = 4$,

47 $$g_{4,4} = - \frac{{3\Gamma \Theta (4k_{T} + 5)}}{{70H(R_{0} ,4)}}\int_{{R_{0} }}^{1} {\frac{{H(\tau ,4)(\tau + E^{ - 1} M)}}{D(\tau ,4)(2\Gamma + \tau )}} d\tau + \frac{4}{{H\left( {R_{0} ,4} \right)}}\int_{{R_{0} }}^{1} {\frac{\tau H(\tau ,4)}{{D(\tau ,4)}}} \overline{g}_{4,4} d\tau ,$$

where

$$H(R_{0} ,n) = \frac{{(R_{0} + 2\Gamma )^{b} }}{{R_{0}^{a} (D(R_{0} ,n))^{c} }},\quad D(R_{0} ,n) = R_{0} + (nk_{T} + n + 1)E^{ - 1} M,$$

in which $a$, $b$ and $c$ are defined as:

$$\begin{aligned} & a = \frac{(n - 1)(n + 2)}{2},\quad b = \frac{{(n - 1)(n + 2)(nk_{T} + n + 1)(E^{ - 1} M - 2\Gamma )}}{{2[(nk_{T} + n + 1)E^{ - 1} M - 2\Gamma ]}}, \\ & c = (n - 1)\frac{{(n + 1 + nk_{T} )E^{ - 1} M - \left( {(n + 1 + nk_{T} )(n + 2) - n} \right)\Gamma }}{{[(nk_{T} + n + 1)E^{ - 1} M - 2\Gamma ]}}. \\ \end{aligned}$$

## References

[1] Sheng HW, Lu K, Ma E (1998). Melting and freezing behavior of embedded nanoparticles in ball-milled Al–10 wt% M (M = In, Sn, Bi, Cd, Pb) mixtures. Acta Mater..

[2] Dippel M (2001). Size-dependent melting of self-assembled indium nanostructures. Phys. Rev. Lett..

[3] Ohashi T, Kuroda K, Saka H (1992). In situ electron microscopy of melting and solidification of in particles embedded in an Fe matrix. Philos. Mag. B.

[4] Saka H, Nishikawa Y, Imura T (1988). Melting temperature of In particles embedded in an Al matrix. Philos. Mag. A.

[5] Sasaki K, Saka H (1991). In situ high-resolution electron microscopy observation of the melting process of In particles embedded in an Al matrix. Philos. Mag. A.

[6] Zhang DL, Cantor B (1990). Heterogeneous nucleation of In particles embedded in an AI matrix. Philos. Mag. A.

[7] Lu K, Jin ZH (2001). Melting and superheating of low-dimensional materials. Curr. Opin. Solid State Mater. Sci..

[8] Lai SL, Carlsson JRA, Allen LH (1998). Melting point depression of Al clusters generated during the early stages of film growth: nanocalorimetry measurements. Appl. Phys. Lett..

[9] Sun J, Simon SL (2007). The melting behavior of aluminum nanoparticles. Thermochim. Acta.

[10] Bachels T, Güntherodt HJ, Schäfer R (2000). Melting of isolated tin nanoparticles. Phys. Rev. Lett..

[11] Mei QS, Lu K (2007). Melting and superheating of crystalline solids: From bulk to nanocrystals. Prog. Mater Sci..

[12] Samsonov VM, Kharechkin SS, Gafner SL, Gafner YY (2009). Molecular dynamics study of the melting and crystallization of nanoparticles. Crystallogr. Rep..

[13] Sdobnyakov NY (2008). On the size dependence of the melting temperature of nanoparticles. Bull. Russ. Acad. Sci. Phys..

[14] Samsonov VM, Vasilyev SA, Bembel AG (2016). Size dependence of the melting temperature of metallic nanoclusters from the viewpoint of the thermodynamic theory of similarity. Phys. Met. Metallogr..

[15] Jiang Q, Zhang S, Zhao M (2003). Size-dependent melting point of noble metals. Mater. Chem. Phys..

[16] Jiang Q, Shi HX, Zhao M (1999). Melting thermodynamics of organic nanocrystals. J. Chem. Phys..

[17] Jiang Q, Shi HX, Zhao M (1999). Free energy of crystal–liquid interface. Acta Mater..

[18] Zhu YF, Lian JS, Jiang Q (2009). Modeling of the melting point, Debye temperature, thermal expansion coefficient, and the specific heat of nanostructured materials. J. Phys. Chem. C.

[19] Xiao BB, Zhu YF, Lang XY, Wen Z, Jiang Q (2014). Al 13@ Pt 42 core-shell cluster for oxygen reduction reaction. Sci. Rep..

[20] Zhu YF, Zhao N, Jin B, Zhao M, Jiang Q (2017). High thermal stability of core–shell structures dominated by negative interface energy. Phys. Chem. Chem. Phys..

[21] McCue SW, Wu B, Hill JM (2009). Micro/nanoparticle melting with spherical symmetry and surface tension. IMA J. Appl. Math..

[22] Back JM, McCue SW, Moroney TJ (2014). Including nonequilibrium interface kinetics in a continuum model for melting nanoscaled particles. Sci. Rep..

[23] Back JM, McCue SW, Hsieh M-N, Moroney TJ (2014). The effect of surface tension and kinetic undercooling on a radially-symmetric melting problem. Appl. Math. Comput..

[24] Duggin MJ (1972). The thermal conductivities of liquid lead and indium. J. Phys. F Metal Phys..

[25] Font F, Myers TG (2013). Spherically symmetric nanoparticle melting with a variable phase change temperature. J. Nanopart. Res..

